# Addressable and unidirectional energy transfer along a DNA three-way junction programmed by pyrrole-imidazole polyamides

**DOI:** 10.1038/srep01883

**Published:** 2013-05-24

**Authors:** Wu Su, Clive R. Bagshaw, Glenn A. Burley

**Affiliations:** 1 Center for Nanomedicine and Nanobiotechnology, Shenzhen Institute of Advanced Technology, Chinese Academy of Sciences, Shenzhen 518055, Guangdong, PR China; 2 Department of Chemistry & Biochemistry, University of California, Santa Cruz, CA 95064, U.S.A; 3 Department of Pure & Applied Chemistry, University of Strathclyde, 295 Cathedral Street, Glasgow, United Kingdom, G1 1XL

## Abstract

We describe a photonic waveguide where FRET is routed uni-directionally along a double-stranded DNA track. The efficiency of FRET is modulated by the supramolecular control of fluorophores along double-stranded DNA using fluorophore-tethered Pyrrole-Imidazole polyamides (PAs). We show that uni-directional FRET is enhanced by the complete assembly of each of the constituent parts, resulting in the selective routing of light along simple DNA duplexes as well as a three-way junction (3WJ).

DNA constitutes a unique scaffold for nanophotonic applications where the organization and manipulation of fluorescence resonance energy transfer (FRET) events can be programmed by the primary sequence of DNA^1^,^2^,^3^,^4^,^5^. The uniqueness of DNA as a bottom-up architecture lies within its ability to self-assemble into sophisticated arrays over micron-scale surface areas^6^,^7^ and with the angstrom-level precision^2^,^8^. The programmability of nanostructures produced by DNA-programmed self-assembly is now finding application in various areas of inter-disciplinary science ranging from protein structure determination^9^, through to new drug delivery vehicles^10^,^11^,^12^. Virtually all of these applications demand interfacing DNA nanostructures with functional materials; whether these are of biological (e.g. proteins) or synthetic (e.g. nanoparticles) origin^2^.

The principal method used to interface DNA nanostructures with non-natural functionality is to prepare modified oligodeoxyribonucleotides (ODNs) by solid phase chemical synthesis^13^,^14^,^15^,^16^,^17^. The assembly of these modified ODNs into sophisticated two- and three-dimensional architectures can then be directed by Watson-Crick base-pairing using either a mixture of ODNs^18^ or a long DNA template (DNA Origami)^19^. Two recent exponents of these approaches are the construction of multi-dimensional light harvesting assemblies and FRET cascades for information transfer and bio-sensing^16^,^20^,^21^,^22^,^23^,^24^,^25^,^26^,^27^,^28^,^29^. These methods highlight the excellent spatial resolution of photonic modules that can be achieved along DNA nanostructures^30^; however this methodology is limited in modularity as once a DNA-programmed array is designed and constructed, the location of specific functional materials or modules are confined to this pre-determined position^1^. An alternative and compatible approach is to utilize a single DNA-programmed nanostructure as the fundamental template and to subsequently append appropriate functionality according to the sequence information within the minor and major grooves of these higher order nanostructures. DNA duplexes are utilized extensively within DNA nanostructures and offer an equivalent level of programmability to traditional Watson-Crick base-pairing used in the construction of conventional DNA nanostructures^31^. For example, the edges of Watson-Crick base-pairs in duplex DNA can be selectively recognized by a commensurate set of pairing rules by small molecules and proteins^32^,^33^,^34^,^35^. A distinct advantage of this rationale is the potential to organize functional materials using un-modified DNA duplexes, thus providing an auxiliary level of self-assembly that is currently underexploited in DNA nanotechnology^36^.

Pyrrole-Imidazole polyamides (PAs) are a family of minor groove binding ligands which have proven ability to organize non-natural functionality along DNA nanostructures with high affinity and selectivity^34^,^35^,^37^. Despite being considerably smaller in molecular weight relative to ODNs, PAs can organize small molecule fluorophores^36^ through to larger molecular weight structures such as proteins^38^,^39^, DNA nanorings^40^,^41^,^42^ and metal nanoparticles^43^. We recently reported the versatility of PAs to construct a DNA-based photonic wire^36^. Using this “PA-programming” approach FRET was observed over 27 nm (80 base pairs) using duplex DNA as the template. Central to this design was a series of homo-FRET processes using oxazole yellow (YO) dyes as FRET “stepping stones”. Since the ET process between the YO dyes is bi-directional, a considerable amount of residual energy resides within the YO dyes rather than being transferred to the final acceptor dye, resulting in ET efficiencies dropping off sharply with increasing numbers of YO-YO ET steps. In order to widen the applicability of this PA-programming approach and develop more sophisticated photonic assemblies and device platforms, a flexible design protocol that controls the position as well as the directionality of each discrete ET step is required^21^.

In this manuscript we describe a novel DNA-based photonic waveguide system where the direction of excitation energy is controlled by the sequence selectivity of DNA-binding PAs. We demonstrate the power and versatility of this approach using a simple DNA model duplex and a three-way junction (3WJ). The routing of photonic energy is observed within these two exemplars by tethering specific fluorophores to two distinct PA sequences (PA**1–3**). We demonstrate that these PAs enable both spatial and spectral manipulation of a uni-directional ET process either down the left or right arms of a 3WJ by the directed assembly of these PAs to binding sites defined to specific locations within the arms of a 3WJ (Fig. 1).

## Results

### Design of uni-directional photonic wire

**Figure 1:**
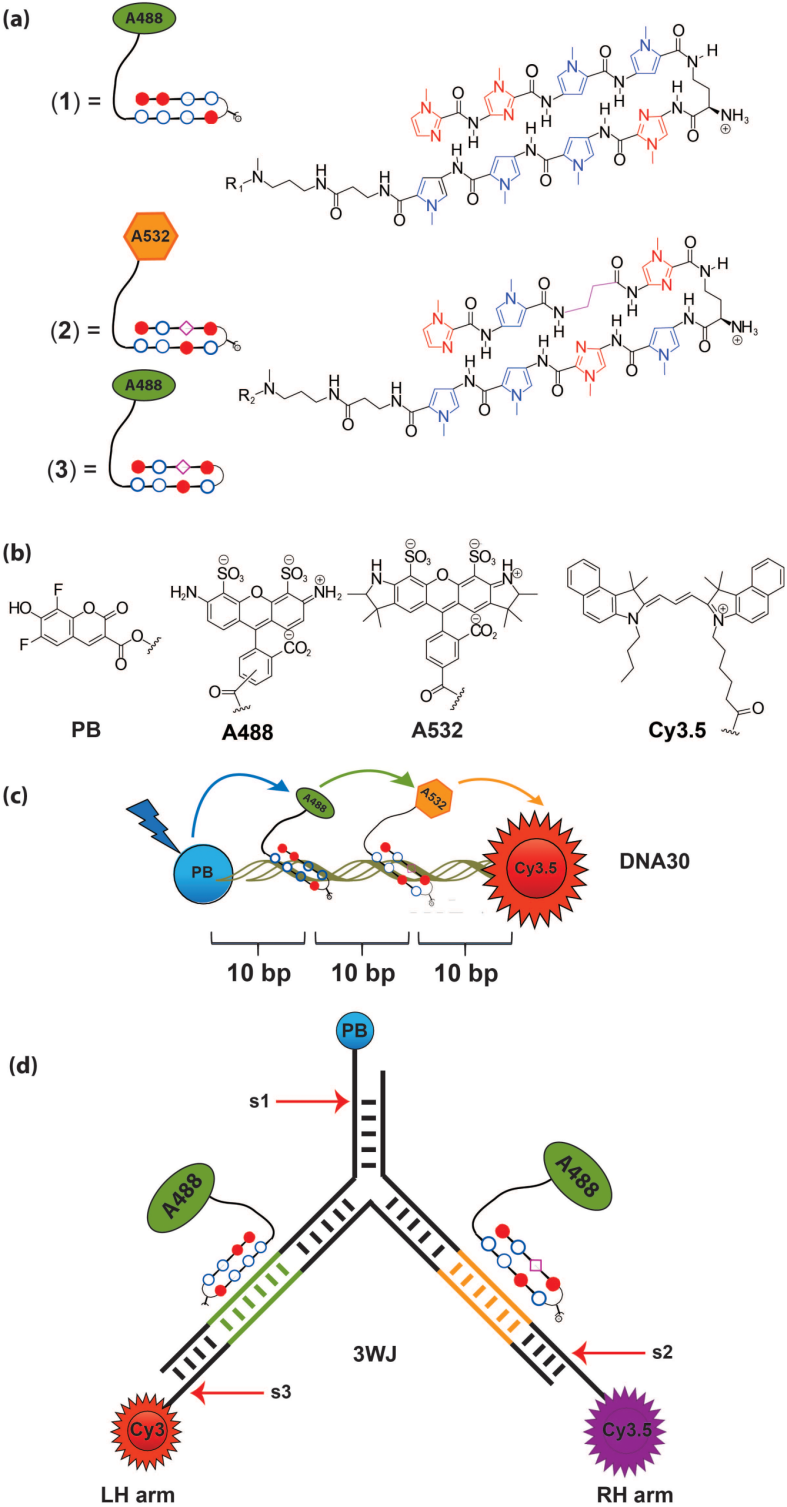
(a) Structures of PAs(1–3). (b) Structures of Pacific Blue (PB, injector), Alexa488, Alexa532 and Cy3.5 (acceptor). (c) Schematic of the exemplar DNA-based photonic wire PBDNA30Cy3.5 and (d) 3WJ used in this study.

*Design of 30-mer DNA duplex (DNA30)* – A 30-mer DNA duplex (PBDNA30Cy3.5) was chosen as our exemplar scaffold in this study (Fig. 1c). This design consisted of two fluorophores: Pacific Blue (PB) as the energy donor and Cyanine 3.5 (Cy3.5) as the corresponding energy acceptor^17^. The target binding sequences for both PA(**1**) and PA(**2**) were also incorporated in this design. These target sequences were separated by ten base-pairs or almost one helical turn of a DNA duplex; placing both fluorophores on the same side of a DNA duplex. This design was chosen in order to maximise dipolar interactions between the two fluorophores.

*Design of the 3WJ* – 3WJ nanostructures are not flat two-dimensional but in fact highly flexible three-dimensional nanostructures which adopt a pyramidal-like structure^44^. A *de novo* designed 3WJ was chosen in this study as it provides one of the simplest three-dimensional exemplars in order to investigate the selectivity of our PA approach. Three fluorescently labelled ODNs (PB-DNA20, Cy3-DNA30, Cy3.5-DNA20) were used to form the 3WJ. The PB energy injector was placed on the 5′ end of the central arm of the 3WJ (s1, Fig. 1d). A 5 base-pair bridge between PB and the central trifurcated branch-point using s2 and s3 was incorporated into the design. A single PA binding site was incorporated on the left hand (LH) and right hand (RH) arm of the 3WJ: the LH arm incorporated a complementary binding sequence (5′-WGGWCW-3′) for PA(**1**) whereas the RH arm contained a complementary binding sequence (5′-WGWCGW-3′) for PA(**3**). Finally the 5′ ends of the LH and RH arms were end-modified with Cy3 and Cy3.5 respectively. We used different energy accepting dyes in our design in order to correlate a specific ET profile to a particular PA-binding event (Fig. 1d).

### Selectivity of PA(1), PA(2) and PA(3) for their target sequences

In order to test whether PA(**1**), PA(**2**) and PA(**3**) were selective for their target sequence, UV-vis melting studies were conducted using dodecamer DNA duplexes (DNA1-2) containing a single PA-binding site (Table 1 and Figures S7–9)^45^,^46^,^47^,^48^. The dodecamer duplex DNA1 contained the binding sequence for PA(**1**) [5′-ATGGACA], whereas duplex DNA2 contained the binding sequence for PA(**2**) and PA(**3**) [5′-ATGACGA]. A 15.9°C stabilization of the duplex melting temperature (T_m_) was observed upon addition of 1.0 equivalent of PA(**1**) to DNA1 to form the complex DNA1@PA(**1**) (Table 1). Only a 2.4°C increase in the T_m_ was observed when 1.0 equivalent of the mismatched PA(**2**) was added to form DNA1@PA(**2**). Conversely PA(**2**) also bound with high affinity and selectivity (ΔT_m_ 12.0°C) to its target 5′-ATGACGA located within duplex DNA2 to form DNA2@PA(**2**). Only a 5.1°C stabilization was observed when the mismatched PA(**1**) was added to DNA2 to form DNA2@PA(**1**). Consistent with T_m _values observed for PA(**2**) when bound to DNA2, PA(**3**) also bound to its target site with high affinity [DNA2@PA(**3**), ΔT_m _10.1°C] compared to DNA1 [DNA1@PA(**3**), ΔT_m _5.3°C] confirming the high binding affinity and selectivity of each PA for their respective target sequences.

### Enhanced fluorescence emission observed upon complete assembly of PA-fluorophore components

**Figure 2:**
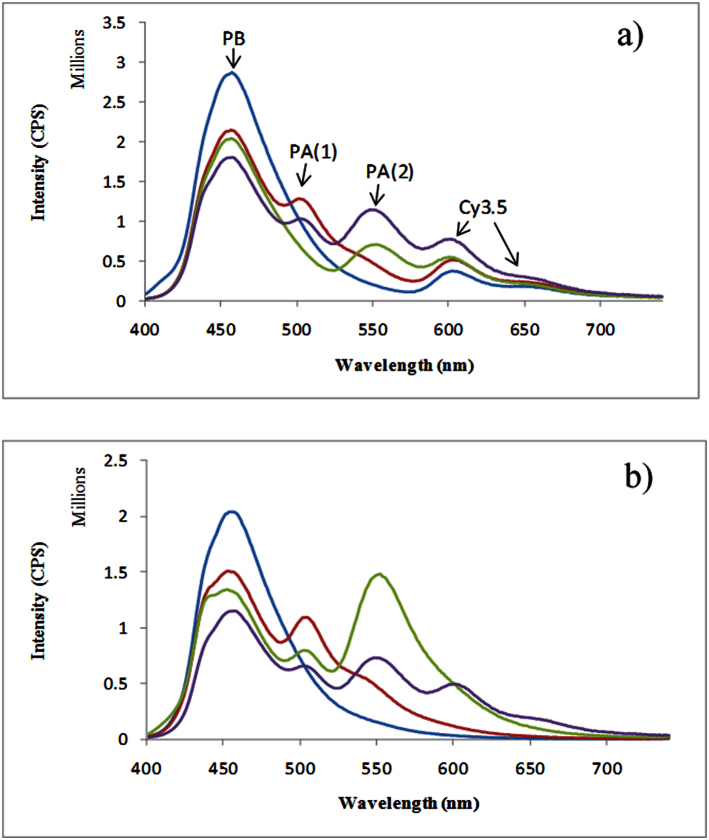
(a) Emission spectra from PB to Cy3.5 of PBDNA30Cy3.5 (blue line), PBDNA30Cy3.5@(**1**) (red line), PBDNA30Cy3.5@(**2**) (green line), PBDNA30Cy3.5@(**1**)@ (**2**), (purple line). (b) Step by step ET of PBDNA30 (blue line), PBDNA30@(**1**) (red line), PBDNA30@(**1**)@(**2**) (green line), PBDNA30Cy3.5@(**1**)@(**2**), (purple line). Excitation was set at 380 nm using a concentration of 100 nM DNA duplex and 100 nM PA(**1**), or/and (**2**).

Steady-state fluorescence emission studies were then undertaken to ascertain whether PAs could facilitate a uni-directional FRET cascade along PBDNA30Cy3.5 (Fig. 2). A slight enhancement of the fluorescence emission of Cy3.5 (λ_em_ 604 nm) was observed in PBDNA30Cy3.5@(**1**) (red line) and PBDNA30Cy3.5@(**2**) (green line) relative to the starting PBDNA30Cy3.5 duplex (blue line). This was indicative of a small degree of ET from the PB injector to the final Cy3.5 acceptor for each of these partially assembled constructs. A 3.5-fold enhancement of the fluorescence emission of Cy3.5 was observed upon complete assembly of all of the components of the photonic wire construct PBDNA30Cy3.5@(**1**)@(**2**) (Fig. 2a & Table 2). To probe the relative contributions of each ET event in the context of the overall ET efficiency, the normalised ET efficiencies were then calculated by deconvolution of the respective emission peaks using a series of photonic wire assemblies (Fig. 2b & Table 3). These measurements confirmed the uni-directional nature of the FRET.

### FRET can be routed selectively along the arms of the 3WJ

Based on the encouraging results using DNA duplexes, a de novo designed 3WJ was chosen to determine if uni-directional FRET events can be routed along the track of a 3WJ. Three fluorescently labelled ODNs (PB-DNA20, Cy3-DNA30, Cy3.5-DNA20) were used to form the 3WJ. The PB energy injector was placed on the 5′ end of the central arm of the 3WJ (Table S1, Fig. 1d). Two PA binding sites were present on the left hand (LH) and right hand (RH) arms of the 3WJ: the LH arm incorporated a complementary binding sequence (5′-WGGWCW-3′) for PA(**1**) whereas the RH arm contained a complementary binding sequence (5′-WGWCGW-3′) for PA(**3**). Finally the 5′ ends of the LH and RH arms were end-modified with Cy3 and Cy3.5 respectively. We used different energy accepting dyes in our design in order to correlate a specific FRET profile to a particular PA-binding event (Fig. 1d). Non-denatured gel electrophoresis confirmed the increased molecular weight of the 3WJ by the difference in electrophoretic mobility of three single DNA strands (PB-DNA20 in Lane 1; Cy3.5-DNA20 in Lane 2; Cy3-DNA30 in Lane 4) relative to the incomplete 3WJ structure (PB-DNA20 with Cy3-DNA30 in Lane 3; PB-DNA20 with Cy3.5-DNA20 and Cy3-DNA30 in Lane 5; Fig. S6).

**Figure 3:**
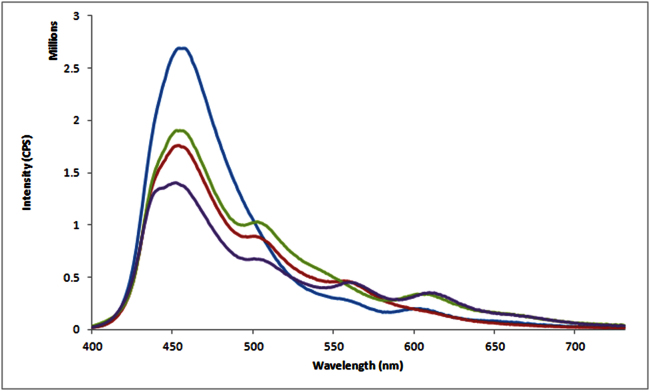
Emission spectra from PB to acceptor Cy3/Cy3.5 along 3WJ exemplar. Emission spectrum from PB to Cy3 and Cy3.5 (blue line); Enhanced ET from PB to Cy3 in 3WJ@(**1**)using PA**1** (i.e. PA-Alexa488) (red line); Enhanced ET from PB to Cy3.5 in 3WJ@(**3**)using PA**3** (i.e. PA-Alexa488) (green line); Enhanced ET from PB to Cy3 and Cy3.5 in 3WJ@(**1**)@(**3**)using PA(**1**)and PA(**3**)(purple line). Excitation was set at 380 nm using a concentration of 100 nM for the 3WJ and 100 nM PA(**1**) or/and (**3**).

Steady-state fluorescence emission studies were then undertaken with our 3WJ assembly (Fig. 3). Excitation of PB in the control 3WJ assembly [i.e. lacking the addition of either PA(**1**) or PA(**3**)] resulted in 11% and 9% of energy transferred to the LH arm (Cy3) and RH arm (Cy3.5) respectively (Fig. 3 & Table 4). Over a three-fold enhancement of FRET down the LH arm of 3WJ@(**1**) was observed upon addition of one equivalent of PA(**1**) (35% ET efficiency from PB to Cy3). A slight increase in the Cy3.5 emission (i.e. from 9% to 12%) was also observed upon addition of PA(**1**) to form 3WJ@PA(**1**), which indicates a small amount of cross-talk (i.e. a 1.33-fold increase in Cy3.5 emission) down the RH arm of 3WJ@(**1**). A similar trend in uni-directional ET was observed in 3WJ@(**3**). In this instance a 2.75-fold enhancement in FRET down the RH arm (i.e. increase in Cy3.5 emission from 12% to 33%) was observed. Concomitantly, a 1.27-fold increase in Cy3 emission (i.e. from 11% to 14%) was observed which paralleled the level of cross-talk observed in the 3WJ@PA(**1**) assembly. The addition of both PA(**1**) and (**3**) to form 3WJ@(**1**)@(**3**) resulted in a 2.5- and 2.6-fold enhancement in FRET down the LH and RH arms of the 3WJ respectively. Deconvolution of each FRET process in the 3WJ nanostructure revealed the second FRET step [i.e. from Alex488 to the final energy acceptor] was efficient as observed by an almost identical loss in PB energy for 3WJ@(**1**) (57%) and 3WJ@(**3**) (56%, Table 4) as well as a minimal amount of residual energy confined to Alex488 [3WJ@(**1**) (8%) and 3WJ@(**3**) (7%)]. Further detailed analysis of the single-step FRET processes revealed that the efficiency from PA(**1**) to Cy3 was 65%, and the efficiency from PA(**3**) to Cy3.5 was 60% (Fig. S11 and Table S2). These single-step data confirmed that the second FRET step were more efficient than the first step.

## Discussion

These experiments were designed to test whether PAs could programme a uni-directional FRET process along a DNA duplex and a 3WJ. Our experiments show that this is indeed the case, however one particular issue that became apparent in our design is the first FRET (i.e. from PB to the PA) step is relatively low. For example, PB → PA(**1**) (23%) and last PA(**2**) → Cy3.5 (28%) FRET steps were considerably lower than the PA(**1**) → PA(**2**) FRET step. Anisotropy measurements of the duplex DNA indicate that the PB and Alexa dyes were freely mobile, whereas the Cy3.5 had restricted motion. It is possible that base stacking of the Cy3.5 dye results in an unfavorable average orientation for efficient FRET, however this would not appear to apply to the other dyes. It is likely that a major contributing factor to the lower than expected FRET efficiency arises from incomplete wire assemblies due to substoichiometric binding and dark states of the dyes (e.g. reversibly or irreversibly photobleaching)^25^,^49^. Single-molecule fluorescence spectroscopy (SMFS) would enable identification and analysis of only complete wires. Indeed SMFS was employed by Tinnefeld *et al.* in a one-dimensional DNA programmed wire assembly^24^,^25^. In their model system, low overall FRET was observed and was attributed to incomplete hybridization of all of the photonic components. The authors highlighted that heterogeneity is considerably reduced by immobilising the photonic wire constructs onto a surface. In completely formed constructs, almost 100% FRET was observed.

The selective enhancement of the fluorescence emission observed down either the LH [i.e. 3WJ@(**1**)] or the RH [i.e. 3WJ@(**3**)] arms of a 3WJ highlights that PAs are effective constructs to recognise DNA duplex sequences embedded within higher order structures. Much akin to our DNA30 example, there is still a significant amount of residual energy confined to the PB injector. Therefore improvements in the choice of injector fluorophore [i.e. replacing PB] will be required in order to increase the end-to-end energy transfer efficiency.

In summary, we report here a DNA-based photonic waveguide platform where spatial and directional FRET is controlled by the molecular recognition of specific duplex sequences within duplex DNA by PAs. FRET can be selectively routed down either the LH or RH arm of a 3WJ and this level of control is achieved by the ability of PA(**1**) and PA(**3**) to target pre-defined binding sequences within this DNA nanostructure. This is the first example of a multi-dimensional DNA-based photonic waveguide where spatial and directional FRET is controlled by duplex DNA acting as a higher order template within a DNA nanoarchitecture. Given the capacity of PAs to recognise sequences up to 16 base pairs in length^50^, PA-programming has the potential to become a powerful complementary technique to organise non-natural functionality along DNA-programmed arrays.

## Methods

### Synthesis of PA(1–3)

Each PA (PA**1–3**) was prepared on a Boc-β-alanine phenylacetamidomethyl (PAM) resin using manual Boc-based solid phase synthetic protocols (Scheme S1 to S3). Cleavage of both resin-bound PA sequences using N1-(3-aminopropyl)-N1-methylpropane-1,3-diamine provided PA(**1b** and **2b**) bearing a primary amine (see Supporting Information for more details). Coupling of the NHS-ester of Alexa488 with PA(**1b**) and PA(**2b**) afforded PA(**1**) and PA(**3**) respectively, whereas coupling of the NHS ester of Alexa532 with PA(**2b**) afforded PA(**2**). The final products were purified by RP-HPLC and characterized by MALDI-MS. Two core PA sequences were used in this study: PA(**1**) which targeted the core sequence 5′-WWGGWCW (W = A/T. The exemplar binding site used in this study was 5′-TAGGACT); PA(**2**) and PA(**3**) which targeted the core sequence 5′ WWGWCGW (The exemplar PA**2**/**3** sequence used in this study was 5′-ATGACGA).

### Steady state fluorescence measurements

Fluorescence measurements were performed using a Horiba Fluorolog 3 fluorimeter. In the dark, 500 μL solutions were prepared in PBS buffer (50 mM Na^+^, pH 7.5), up to a final concentration of 100 nM for the DNA duplex and 100 nM (**1**), (**2**) or/and (**3**). The solutions were gently shaken and then allowed to sit at room temperature for 0.5 h. The measured sample was placed in a 500 μL quartz cell with 5 mm path length and kept at 20°C during the measurement. Corrected emission spectra were collected from 400 nm to 730 nm using an excitation wavelength of 380 nm.

## Author Contributions

W.S. performed the experiments, contributed to the interpretation of the experimental data and contributed to the writing of the manuscript. C.R.B. contributed to the interpretation of the experimental data and contributed to the writing of the manuscript. G.A.B. contributed to the interpretation of the experimental data and wrote the manuscript.

## Supplementary Material

Supplementary Information Supporting Information

## Figures and Tables

**Table 1:** UV-vis melting temperatures of dsDNA1 and dsDNA2 in the absence and presence of PA (1–3)

	Tm (°C)	ΔT_m_(°C)
DNA1	51.6	-
5′- CT**A TGGACA** AGC- 3′		
3′- GA**TACCTGT** TCG- 5′		
DNA2	51.2	-
5′- CT**ATGACGA** AGC - 3′		
3′- GA**TACTGCT** TCG - 5′		
PA(**1**) + DNA1	67.5	15.9
PA(**1**) + DNA2	53.6	2.4
PA(**2**) + DNA1	56.7	5.1
PA(**2**) + DNA2	63.2	12.0
PA(**3**) + DNA1	56.9	5.3
PA(**3**) + DNA2	61.3	10.1

**Table 2:** Calculated end-to-end ET efficiencies for DNA30 assemblies

DNA construct	End-to-end ET efficiency (%)
PBDNA30Cy3.5	4
PBDNA30Cy3.5@(**1**)	8
PBDNA30Cy3.5@(**2**)	8
PBDNA30Cy3.5@(**1**)@(**2**)	14

**Table 3:** Estimated donor energy losses and acceptor sensitized emission (em) efficiencies in selected DNA wires

	PB loss	PA (1) em	PA (2) em	Cy3.5 em
PBDNA30	0%	-	-	-
PBDNA30@(**1**)	25%	18%	-	-
PBDNA@(**1**)@(**2**)	32%	7%	19%	-
DNA30Cy3.5@(**1**)@(**2**)	42%	5%	16%	14%

**Table 4:** Estimated donor energy losses and acceptor sensitized emission (em) efficiencies in 3WJ assemblies

DNA construct	PB loss (%)	Alex488 (%) emission	Cy3 (%) emission	Cy3.5 (%) emission
3WJ	27	0	11	9
3WJ@(**1**)	57	8	35	12
3WJ@(**3**)	56	7	14	33
3WJ@(**1**)@(**3**)	63	6	28	24
