# Supplementary material for: Addressable and unidirectional energy transfer along a DNA three-way junction programmed by pyrrole-imidazole polyamides
Source: Sci Rep. 2013 May 24;3:1883. doi: 10.1038/srep01883 (PMC3662969; doi:10.1038/srep01883)
Supplement: Supplementary Information — Supporting Information [file srep01883-s1.doc]

Addressable and uni-directional energy transfer along a DNA three-way junction programmed by pyrrole-imidazole polyamides

Wu. Su,^†^ Clive R. Bagshaw^‡^ and Glenn A. Burley^*, §^

^†^ Center for Nanomedicine and Nanobiotechnology, Shenzhen Institutes of Advanced Technology, Chinese Academy of Sciences, Shenzhen 518055, Guangdong, PR China.

‡ Department of Chemistry & Biochemistry, University of California, Santa Cruz, CA 95064, U.S.A.

^§^ Department of Pure & Applied Chemistry, University of Strathclyde, 295 Cathedral Street, Glasgow, G1 1XL, UK.

Table of Contents

[Addressable and uni-directional energy transfer along a DNA three-way junction programmed by pyrrole-imidazole polyamides 1](#__RefHeading___Toc332193204)

[1.0 Abbreviations 2](#__RefHeading___Toc332193205)

[2.0 Experimental Section 2](#__RefHeading___Toc332193206)

[2.1 General. 2](#__RefHeading___Toc332193207)

[2.2 Synthesis of polyamide conjugate (1), (2) and (3). 3](#__RefHeading___Toc332193208)

[2.3 DNA duplex or triplex preparation. 9](#__RefHeading___Toc332193209)

[2.4 UV absorption spectrophotometry. 10](#__RefHeading___Toc332193210)

[2.5 Steady state Fluorescence Measurements 12](#__RefHeading___Toc332193211)

[2.6 Calculation of energy transfer efficiencies 12](#__RefHeading___Toc332193212)

[3.0 References 16](#__RefHeading___Toc332193213)

1.0 Abbreviations

BTC, Bis-(trichlormethyl)-carbonate; DCM, dichloromethane; DIEA, N-ethyldiisopropylamine; DMF, N, N’-dimethylformamide; Fmoc-D-Dab(Boc)-OH, N-α-(9-fluorenylmethyloxycarbonyl)-N-γ-tbutyloxycarbonyl-D-2,4-diaminobutyric acid; HATU, 2-(1H-7-Azabenzotriazol-1-yl)-1,1,3,3-tetramethyl uronium hexafluorophosphate; NBS, N-bromosuccinimide; TEA, triethylamine; TFA, Trifluoroacetic acid; THF, Tetrahydrofuran.

2.0 Experimental Section

### 2.1 General

All reagents were either HPLC or peptide synthesis grade. DMF, DCM, TFA, THF and DCC were obtained from Acros Organics. BTC, DIEA, and DMPA were purchased from Sigma-Aldrich. Boc-β-Ala-PAM resin was purchased from Peptides International (UK). Fmoc-D-Dab(Boc)-OH was purchased from ABCR. 1-methyl-1H-imidazole-2-carboxylic acid was purchased from Maybridge Chemicals. 5’- PB labelled DNA strands, Alexa 488-NHS ester and Alexa 532-NHS ester were purchased from Invitrogen. Non-labelled and 5’- Cy3 or Cy3.5 labelled DNA strands were purchased from Eurogentec.

Boc-Py-OH and Boc-Im-OH were prepared according to literature procedures.^[1]^

Analytical and semipreparative RP-HPLC was performed at room temperature on the ULTIMAT 3000 Instrument (DIONEX). UV absorbance was measured using a photodiode array detector at 260 and 310nm. An ACE C18 column (4.6 X 250 mm, 5 μm, 300 Å) was used for analytical RP-HPLC. For semipreparetive HPLC, an ACE C18 column (10 X 250 mm, 5 μm, 300 Å) was used. MALDI-MS was performed on ion trap SL 1100 system (Agilent).

### 2.2 Synthesis of polyamide conjugate (1), (2) and (3)

PAs (**1a**) and (**2a**) were synthesized by solid phase synthesis on a Boc-β-Ala-PAM resin (200 mg) at a substitution level of 0.15 mmol/g using a synthetic methodology previously reported by our laboratory.^[2]^ After solid phase synthesis, the polyamide bonded resin (**1a**) and (**2a**) was treated with 2 mL 3,3’-Diamino-N-methyl-dipropylamine (**3**) at 55**°**C for 18 h. The reaction mixture was filtered to remove resin. The filtrates were dried under vacuum. The light-yellow powders were dissolved in DMSO/H_2_O (30/70 v/v) and purified by semi-preparative reverse phase HPLC (semi-preparative HPLC gradient: started at 10% of B (0.1 TFA in MeCN), hold on 5 min, then increased to 60% of B in 25 min). Lyophilization of the products afforded fine yellow powder (For **1b:** 5.7 mg, 14% recovery; For **2b:** 6.3 mg, 16% recovery). PAs (**1b**) and (**2b**) were characterized by MALDI-MS (monoisotopic): For **1b:** calcd m/z 1381.5, found m/z 1381.5 (Figure S1) ; For **2b:** calcd m/z 1330.5, found m/z 1330.4 (Figure S2).

**Im-Im-Py-Py-γ(NH_2_)-Im-Py-Py-Py-β-DP-Alexa488 (1)** (Scheme-S1)**.** Polyamide **1b** (1 µmol aliquot) was dissolved in 500 µL of DMSO, treated with Alexa488-NHS ester (4) (2 eq), and DIEA (20 µL), at room temperature for 2 h. Next, the mixture was treated with TFA (500 µL) for 5 min. After then, 10 mL diether added to precipitate the crude product. After centrifuge, the crude product was dissolved in DMSO/H_2_O (30/70 v/v) and purified by semi-preparative reverse phase HPLC. Lyophilization of the products afforded fine orange powder **(1)** (1.2 mg, 67% recovery). PA (**1**) was characterized by MALDI-MS (monoisotopic): calcd m/z 1797.9, found m/z 1798.1 (Figure S3).

**Im-Py-β-Im-γ(NH_2_)-Py-Im-Py-Py-β-DP-Alexa532 (2)** (Scheme-S2)**.** Polyamide **2b** (1 µmol aliquot) was dissolved in 500 µL of DMSO, treated with Alexa532-NHS ester (5) (2 eq), and DIEA (20 µL), at room temperature for 2 h. Next, the mixture was treated with TFA (500 µL) for 5 min. After then, 10 mL diether added to participate the crude product. After centrifuge, the crude product was dissolved in DMSO/H_2_O (30/70 v/v) and purified by semi-preparative reverse phase HPLC. Lyophilization of the products afforded fine orange powder **(2)** (1.1 mg, 58% recovery). PA (**2**) was characterized by MALDI-MS (monoisotopic): calcd m/z 1883.0, found m/z 1883.4 (Figure S4).

**Im-Py-β-Im-γ(NH_2_)-Py-Im-Py-Py-β-DP-Alexa488 (3)** (Scheme-S3)**.** Polyamide **2b** (1 µmol aliquot) was dissolved in 500 µL of DMSO, treated with Alexa488-NHS ester (4) (2 eq), and DIEA (20 µL), at room temperature for 2 h. Next, the mixture was treated with TFA (500 µL) for 5 min. After then, 10 mL diether added to participate the crude product. After centrifuge, the crude product was dissolved in DMSO/H_2_O (30/70 v/v) and purified by semi-preparative reverse phase HPLC. Lyophilization of the products afforded fine orange powder **(3)** (1.4 mg, 67% recovery). PA (**3**) was characterized by MALDI-MS (monoisotopic): calcd m/z 1839.1, found m/z 1839.5 (Figure S5).

***Figure S1****. MALDI-ToF mass spectrum of polyamide (****1b****).*

***Figure S2.*** *MALDI-ToF mass spectrum of polyamide (****2b****).*

***Figure S3.*** *MALDI-ToF mass spectrum of polyamide-Alexa 488 conjugate (****1****).*

***Figure S4.*** *MALDI-ToF mass spectrum of polyamide-Alexa 532 conjugate (****2****).*

***Figure S5.*** *MALDI-ToF mass spectrum of polyamide-Alexa 488 conjugate (****3****).*

***Scheme S1.*** *Synthesis of polyamide-Alexa 488 conjugate (****1****).*

***Scheme S2.*** *Synthesis of polyamide-Alexa 532 conjugate (****2****).*

***Scheme S3.*** *Synthesis of polyamide-Alexa 488 conjugate (****3****).*

### 2.3 DNA duplex or triplex preparation.

Equimolar amounts of fluorophore-labelled (or identical non-labelled control DNA sequence) DNA strands were mixed in sodium phosphate buffer (PBS, 50 mM Na^+^, pH7.5) at room temperature to a concentration of 10 µM double strand DNA. Annealing was performed by heating the samples to 85 °C for 10 min, followed by slow cooling to 25 °C over a 6 h period.

***Table S1:*** *DNA sequences used in this study. Bold-faced base pairs correspond to PA binding sites.*

| Sequence name | Sequence | Note |
| --- | --- | --- |
| DNA30 | 5’ PB- CAT AGT ATA TA**T** **GGA CT**A TAT **AGA CGA** ATC 3’  3’ GTA TCA TAT AT**A CCT GA**T ATA **TCT GCT** TAG-Cy3.5 5’ | 1D photonic wire |
| DNA30_Control | 5’- CAT AGT ATA TA**T** **GGA CT**A TAT **AGA CGA** ATC 3’  3’ GTA TCA TAT AT**A CCT GAT** ATA **TCT GCT** TAG-Cy3.5 5’ | 1D photonic wire without PB |
| YJ_DNA | 5’ Cy3- GAT **AGT CCT** ATC TCT TGT GAT **TGA CGT** TAG 3’  3’ CTA **TCA GGA** TAG AGA ACA CTA **ACT GCA** ATC-Cy3.5 5’  T A  G C  T A  C G  G C  I 3’  5’  PB | 3WJ- photonic wire |
| YJ_DNA_Control | 5’ Cy3- GAT **AGT CCT** ATC TCT TGT GAT **TGA CGT** TAG 3’  3’ CTA **TCA GGA** TAGAGA ACA CTA **ACT GCA** ATC-Cy3.5 5’  T A  G C  T A  C G  G C  5’ 3’ | 3WJ-photonic wire without PB |

### **
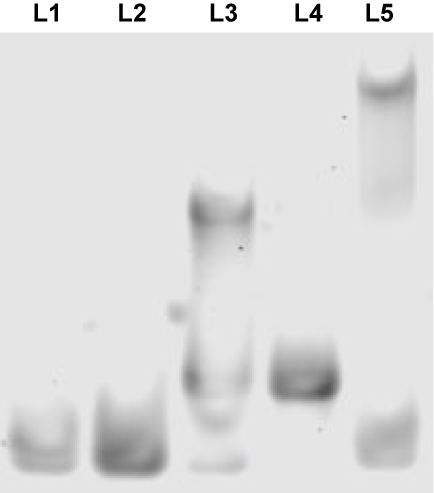
**

***Figure S6.*** *15% Polyacrylamide native gel electrophoresis of the 3WJ used in this study. L1: PBDNA20; L2: Cy3.5DNA20; L3: PBDNA20 + Cy3DNA30; L4: Cy3DNA30; L5: 3WJ (i.e. PBDNA20 + Cy3DNA30 + Cy3.5DNA20.*

### 2.4 UV absorption spectrophotometry.

Melting temperature analysis was performed on a PerkinElmer Lambda 35 UV/Vis spectrophotometer equipped with a thermo-controlled cell holder possessing a cell path length of 1 cm. A degassed aqueous solution of 10 mM sodium cacodylate, 10 mM KCl, 10 mM MgCl_2_, and 5 mM CaCl_2_ at pH 7.0 was used as the analysis buffer.^[3]^ The corresponding DNA duplex (dsDNA1: 5’- CTA T*GG AC*A AGC- 3’ with complementary DNA sequence; dsDNA2: 5’- CTA T*GA CG*A AGC - 3’ with complementary DNA sequence), compounds (1), (2) or (3) were mixed with 1:1 stoichiometry to a final concentration of 1 µM for each experiment. Prior to analysis, samples were heated to 90 °C, and cooled to a starting temperature of 25 °C with a heating rate of 5 °C/min for each ramp. Denature profiles were recorded at λ = 260 nm from 35 to 85 °C (Figure S7-9). The reported melting temperatures were defined as the maximum of the first derivative of the denature profile.


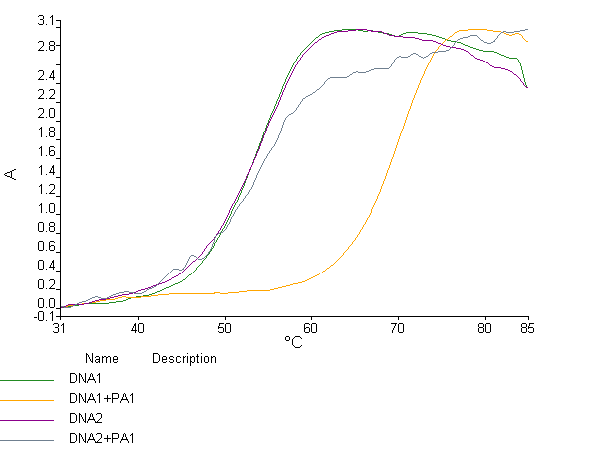

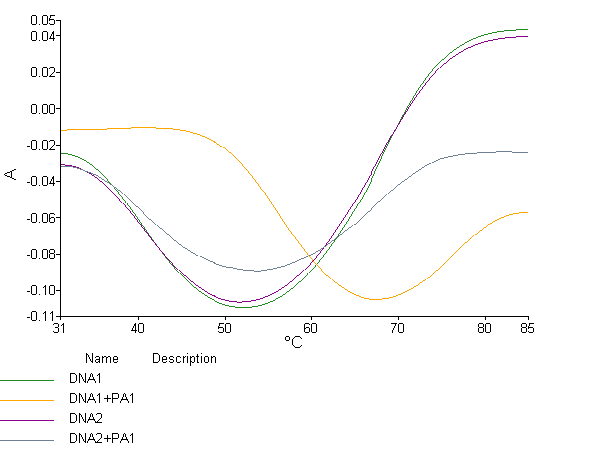


***Figure S7.*** *Normalised UV isothermal binding profiles and the first derivative of the denature profile of DNA12 duplex in the absence and presence of PA488 (****1****).*


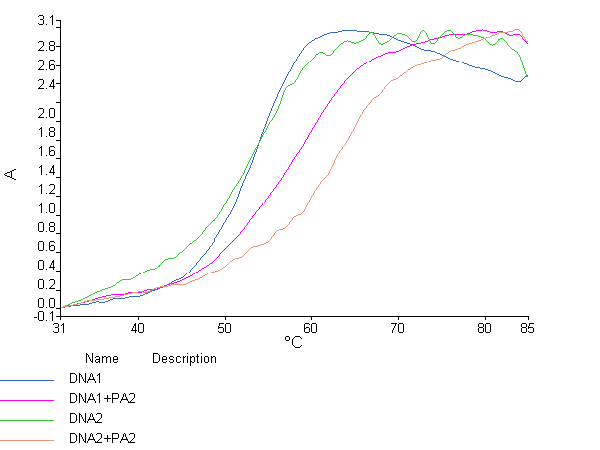

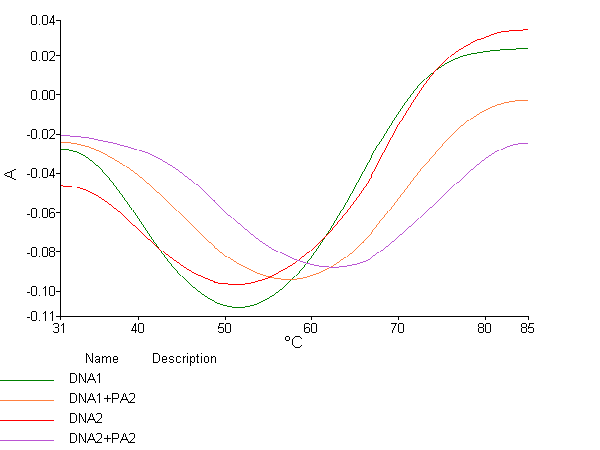


***Figure S8.*** *Normalised UV isothermal binding profiles and the first derivative of the denature profile of DNA12 duplex in the absence and presence of PA532 (****2****).*


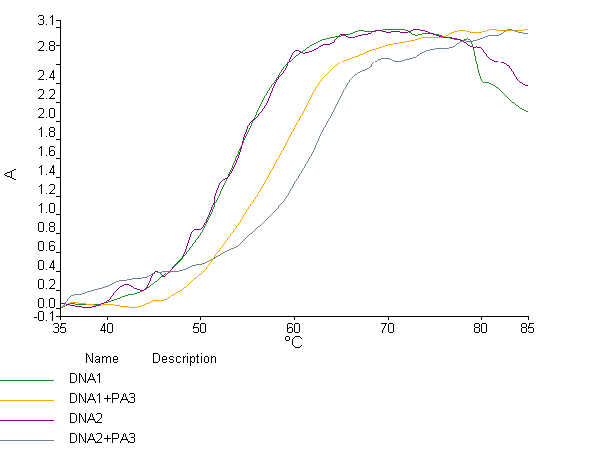

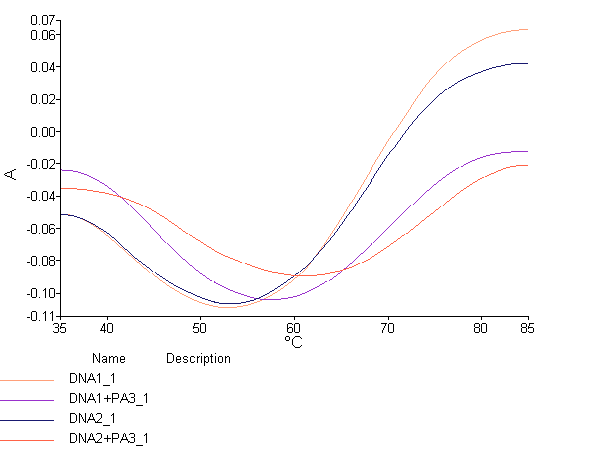


***Figure S9.*** *Normalised UV isothermal binding profiles and the first derivative of the denature profile of DNA12 duplex in the absence and presence of PA488 (****3****).*

### 2.5 Steady state Fluorescence Measurements

Steady state fluorescence measurements were performed using a Horiba Fluorolog 3 fluorimeter. In the dark, 500 µL solutions were prepared in PBS buffer (50 mM Na^+^, pH7.5), up to a final concentration of 100 nM for the DNA duplex and 100 nM (**1**), (**2**) or/and (**3**). The solutions were gently shaken and then allowed to sit at room temperature for 0.5 h. The measured sample was placed in a 500 µL quartz cell with 5 mm path length and kept at 20 °C during the measurement. Corrected emission spectra were collected from 400 nm to 730 nm using an excitation wavelength of 380 nm.

### 2.6 Calculation of energy transfer efficiencies

To compare the transfer efficiency of the wires, the number of photons emitted from PB, Alexa488, Alexa532, Cy3 and Cy3.5 were deduced from the wire spectra by reconstructing the wire spectra through a linear combination of standard spectra of the individual chromophores: For DNA30 linear assembly: W(λ)=A*PB(λ)+B*Alex488(λ)+C*Alex532(λ)+D*Cy3.5(λ); for 3WJ assembly: W(λ)=A*PB(λ)+B*Alex488(λ)+C*Cy3(λ)+D*Cy3.5(λ). The pre-factors A, B, C and D were determined from least-square fitting routines and indicate the relative contributions of the individual components to the wire spectra.

Energy transfer efficiencies were calculated according to Hannestad *et al.*^[4]^ In brief, efficiencies were calculated according to eq. S1. This analysis is applied to both the terminal acceptors and intermediary acceptors following deconvolution and correction of the direct excitation component. Dividing the extracted acceptor emission with the fluorescence quantum yield of acceptor, the total excitation energy transferred from the PB is obtained. The unquenched PB emission is divided by the PB quantum yield to afford the energy of initially excited PB molecules (eq. S1).


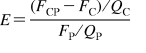


*Equation S1: Energy efficiency calculation. F­_CP_ represents the integrated acceptor emission spectra (wavenumber scale) (excitation at 380 nm) with PB. F­_C_ represents the integrated acceptor emission spectra (wavenumber scale) (excitation at 380 nm) without PB. Q­_C_ represents the quantum yield of acceptor. Fp is the integrated donor (PB) emission without acceptors. Q­_P_ represents the quantum yield of PB-labelled DNA.*

2.7 FRET analysis along 3WJ

**(a)**

**
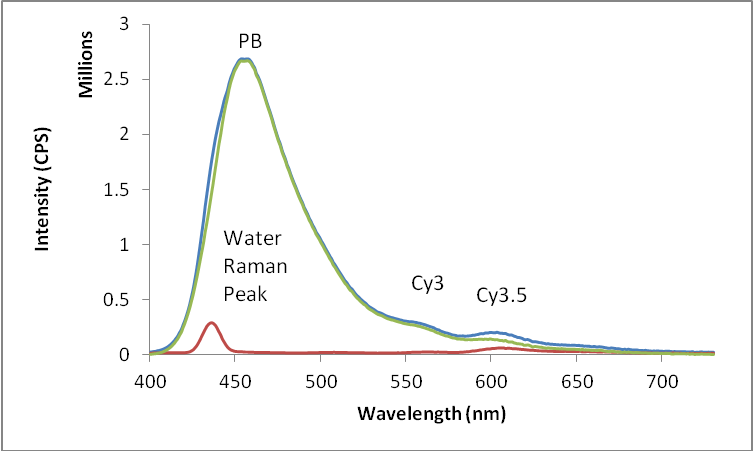
**

**(b)**

**
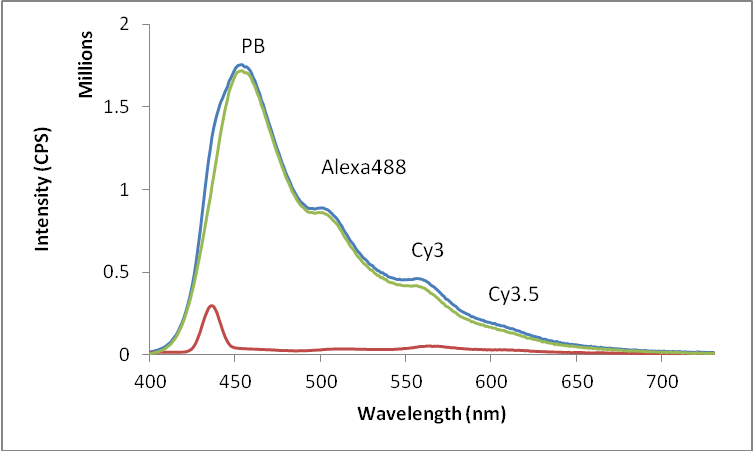
**

**(c)**

**
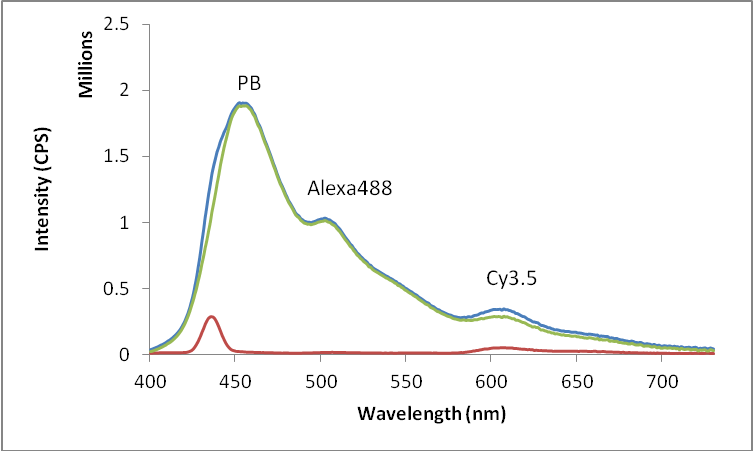
**

**(d)**


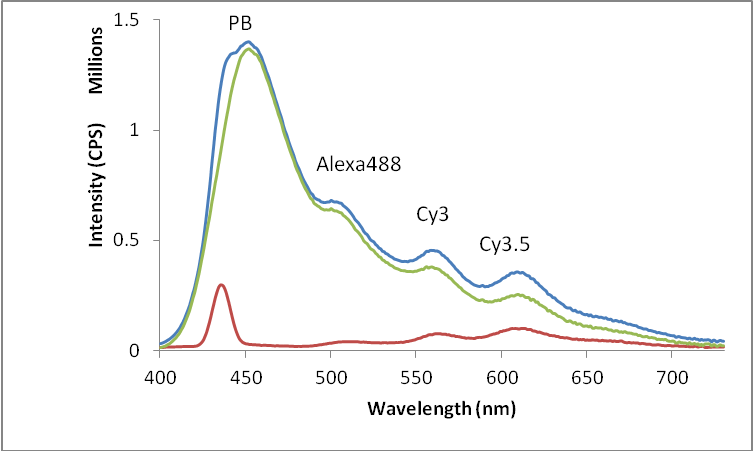


***Figure S10.*** *Emission spectra from PB to acceptor dyes Cy3/Cy3.5 along 3WJ exemplar. (a) Emission spectrum from PB to Cy3 and Cy3.5 (blue line); direct excitation of Cy3 and Cy3.5 (red line); and normalized emission spectrum from PB to Cy3 and Cy3.5 (green line). (b) Enhanced ET from PB to Cy3 in 3WJ@(1) using PA1 (i.e. PA-Alexa488) (blue line); direct excitation of Alexa488, Cy3 and Cy3.5 (red line);and normalized emission spectrum from PB to Cy3 (green line). (c) Enhanced ET from PB to Cy3.5 in 3WJ@(3) using PA3 (i.e. PA-Alexa488) (blue line); direct excitation of Alexa488, Cy3 and Cy3.5 (red line); and normalized emission spectrum from PB to Cy3.5 (green line). (d) Enhanced ET from PB to Cy3 and Cy3.5 in 3WJ@(1)@(3) using PA(1) and PA(3) (blue line); direct excitation of Cy3 and Cy3.5 (red line); and normalized emission spectrum from PB to Cy3 and Cy3.5 by PA1 and PA3 (green line). Excitation was recorded at 380 nm using a concentration of 100 nM for the 3WJ and 100 nM PA(1) or (3); direct excitations (red lines in a, b, c, and d) indicated the excitations were  recorded at 380 nm from the constructs lacking donor PB.*


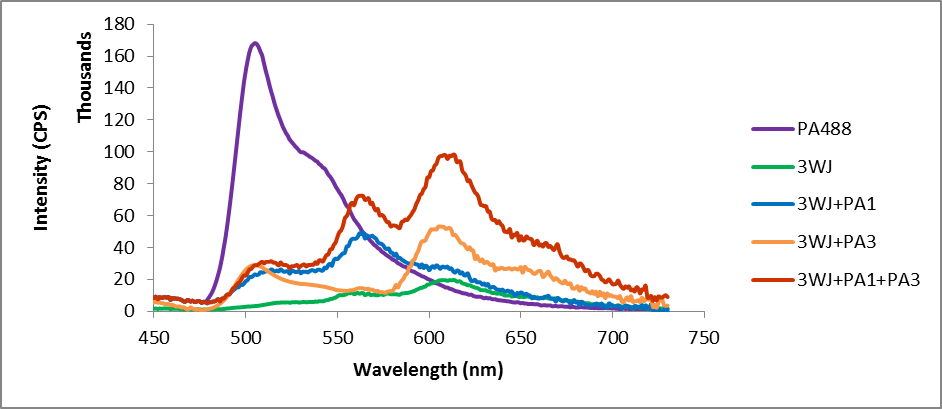


***Figure S11.*** *Emission spectra from PA(****1****) and/or PA(****3****) to acceptor Cy3/Cy3.5 along 3WJ (without Pacific Blue) exemplar. i) Emission spectrum of PA(****1****) or PA(****3****) (PA488, purple line); ii) Emission spectrum of 3WJ with Cy3 and Cy3.5 (green line); iii) Enhanced FRET from PA(****1****) to Cy3 in 3WJ@(****1****) (i.e. PA(****1****)-Alexa488) (blue line); iv) Enhanced FRET from PA(****3****) to Cy3.5 in 3WJ@(****3****) (i.e. PA(****3****)-Alexa488) (orange line); v) Enhanced FRET from PA(****1****) and PA(****3****) to Cy3 and Cy3.5 in 3WJ@(****1****)@(****3****)using PA(****1****)and PA(****3****)(red line). Excitation was set at 380 nm using a concentration of 100 nM for the 3WJ and 100 nM PA(****1****) or/and (****3****).*

***Table S2****. Estimated donor PA(****1****) and/or PA(****3****) energy losses and acceptor sensitized emission (em) efficiencies in 3WJ assemblies (without Pacific Blue).*

| **DNA construct** | **PA(1) loss (%)** | **PA(3) loss (%)** | **Cy3 (%)**  **emission** | **Cy3.5 (%)**  **emission** |
| --- | --- | --- | --- | --- |
| 3WJ@(**1**)  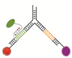 | 83 |  | 65 | 11 |
| 3WJ@(**3**)  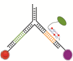 |  | 76 | 14 | 60 |
| 3WJ@(**1**)@(**3**)  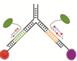 | PA(**1**)+PA(**3**) =81×2 | | 74 | 69 |

3.0 References

[1] E. E. Baird, P. B. Dervan, *Journal of the American Chemical Society* **1996**, *118*, 6141.

[2] W. Su, S. J. Gray, R. Dondi, G. A. Burley, *Organic Letters* **2009**, *11*, 3910.[3] C. Dose, M. E. Farkas, D. M. Chenoweth, P. B. Dervan, *Journal of the American Chemical Society* **2008**, *130*, 6859.

[4] J. K. Hannestad, P. Sandin, B. Albinsson, *Journal of the American Chemical Society* **2008**, *130*, 15889.
